# Linoleic acid improves assembly of the CII subunit and CIII2/CIV complex of the mitochondrial oxidative phosphorylation system in heart failure

**DOI:** 10.1186/s12964-019-0445-0

**Published:** 2019-10-16

**Authors:** Satoshi Maekawa, Shingo Takada, Hideo Nambu, Takaaki Furihata, Naoya Kakutani, Daiki Setoyama, Yasushi Ueyanagi, Dongchon Kang, Hisataka Sabe, Shintaro Kinugawa

**Affiliations:** 1 Department of Cardiovascular Medicine, Hokkaido University Graduate School of Medicine, Kita-15, Nishi-7, Kita-ku, Sapporo, 060-8638 Japan; 2 Department of Molecular Biology, Hokkaido University Graduate School of Medicine, Sapporo, Japan; 3 Faculty of Lifelong Sport, Department of Sports Education, Hokusho University, Ebetsu, Japan; 4 Research Fellow of the Japan Society for the Promotion of Science, Tokyo, Japan; 5 Clinical Laboratories, Kyushu University Hospital, Fukuoka, Japan; 6 Department of Clinical Chemistry and Laboratory Medicine, Graduate School of Medical Sciences, Kyushu University, Fukuoka, Japan

**Keywords:** Supercomplex, Myocardial infarction, Cardiolipin, Succinate dehydrogenase, Respirasome

## Abstract

**Background:**

Linoleic acid is the major fatty acid moiety of cardiolipin, which is central to the assembly of components involved in mitochondrial oxidative phosphorylation (OXPHOS). Although linoleic acid is an essential nutrient, its excess intake is harmful to health. On the other hand, linoleic acid has been shown to prevent the reduction in cardiolipin content and to improve mitochondrial function in aged rats with spontaneous hypertensive heart failure (HF). In this study, we found that lower dietary intake of linoleic acid in HF patients statistically correlates with greater severity of HF, and we investigated the mechanisms therein involved.

**Methods:**

HF patients, who were classified as New York Heart Association (NYHA) functional class I (*n* = 45), II (*n* = 93), and III (*n* = 15), were analyzed regarding their dietary intakes of different fatty acids during the one month prior to the study. Then, using a mouse model of HF, we confirmed reduced cardiolipin levels in their cardiac myocytes, and then analyzed the mechanisms by which dietary supplementation of linoleic acid improves cardiac malfunction of mitochondria.

**Results:**

The dietary intake of linoleic acid was significantly lower in NYHA III patients, as compared to NYHA II patients. In HF model mice, both CI-based and CII-based OXPHOS activities were affected together with reduced cardiolipin levels. Silencing of *CRLS1*, which encodes cardiolipin synthetase, in cultured cardiomyocytes phenocopied these events. Feeding HF mice with linoleic acid improved both CI-based and CII-based respiration as well as left ventricular function, together with an increase in cardiolipin levels. However, although assembly of the respirasome (i.e., CI/CIII_2_/CIV complex), as well as assembly of CII subunits and the CIII_2_/CIV complex statistically correlated with cardiolipin levels in cultured cardiomyocytes, respirasome assembly was not notably restored by dietary linoleic acid in HF mice. Therefore, although linoleic acid may significantly improve both CI-based and CII-based respiration of cardiomyocytes, respirasomes impaired by HF were not easily repaired by the dietary intake of linoleic acid.

**Conclusions:**

Dietary supplement of linoleic acid is beneficial for improving cardiac malfunction in HF, but is unable to completely cure HF.

## Background

The prevalence of heart failure (HF) has increased globally, particularly in developed countries in recent decades [1]. Advances in our understanding of the underlying pathophysiologic mechanisms have given rise to new therapies for treating the increasing population with HF. Nonetheless, morbidity and mortality associated with HF and its financial implications remains a large concern. Thus, novel therapies that can improve the natural history of HF patients are urgently needed [2]. Likewise, what actually constitutes a ‘balanced diet’ that can prevent as well as treat HF should be reconsidered, together with identification of the molecular mechanisms therein involved.

An individual’s daily diet can affect the severity of HF [3]. However, only fluid and salt restriction are recommended regarding diet therapy for HF patients. Fats, which are essential nutrients, are absorbed by the body and subsequently decomposed into fatty acids, which are components of energy substrates and plasma membranes (i.e., cells, nuclei, and mitochondria) [4, 5]. Linoleic acid (18:2), an essential fatty acid, is a major component of cardiolipin, which is a specific mitochondrial phospholipid. Mulligan et al. reported that moderate consumption of linoleic acid in aged spontaneous hypertensive HF rats (Mcc^facp−/−^) prevented the reduction of cardiolipin content and the exacerbation of mitochondrial function [6], but its mechanism remains unknown.

Structural studies have shown that cardiolipin is important for stabilization (i.e., the assembly of complex subunits) of mitochondrial oxidative phosphorylation (OXPHOS) complex I (CI) in sheep, [7] complex II (CII) in *S. cerevisiae* (in the absence of CI), [8] complex III (CIII) in cows, [9] and complex IV (CIV) in *S. cerevisiae* [10]. Although a decrease in cardiolipin levels promotes the disassembly of these mitochondrial OXPHOS complexes, it has not yet been clearly demonstrated whether dietary intake of linoleic acid can restore the integrity of these complexes as well as cardiac dysfunction.

We here found that HF patients with greater pathological severity statistically take lower amounts of dietary linoleic acid compared with HF patients with lower severity. Consistent with a previous report of an animal experiment, [6] we then found that linoleic acid can improve cardiolipin levels that were otherwise reduced in cardiac mitochondria of HF model mice. Nevertheless, our study also suggests that linoleic acid supplementation cannot fully restore the mitochondrial respirasome in HF mice. Together with the molecular bases by which linoleic acid improves the OXPHOS activity of cardiac mitochondria, as well as left ventricular function, our study suggests that insufficient intake of linoleic acid might be associated with HF.

## Methods

### Patients with HF

A multicenter and prospective cohort study investigating the effect of malnutritional status on clinical outcomes in patients with chronic HF was performed. All the enrolled patients treated at 10 institutes ((1) Department of Nutritional Management Center, Hokkaido University Hospital; (2) Department of Cardiology, Kitami Red Cross Hospital; (3) Department of Molecular Medicine and Therapeutics, Tottori University Faculty of Medicine; (4) Department of Cardiovascular Medicine, Kushiro City General Hospital; (5) Department of Cardiology, Keiwakai Ebetsu Hospital; (6) Division of Cardiology, Cardiovascular and Aortic Center of Saiseikai Fukuoka General Hospital; (7) Hikone Municipal Hospital; (8) Otaru Kyokai Hospital; (9) National Hospital Organization Hospital, Hokkaido; (10) Hokkaido Social Work Association Obihiro Hospital) provided their written informed consent to participate in this study. The study was approved by the institutional ethics committee at each participating site and was conducted in accordance with the ethical principles described in the Declaration of Helsinki (2013 revised version).

Patient eligibility criteria were as follows: age ≥ 20 years; one or more hospitalizations owing to worsening HF before enrollment; outpatients with stable New York Heart Association (NYHA) functional class I, II, or III for longer than 1 month since their prior hospitalization; and available written informed consent of the patient. Patients were excluded if they had nephrotic syndrome, liver cirrhosis, cancer, a history of gastrointestinal surgery within the previous 3 months, or poorly controlled diabetes (hemoglobin A1c > 7.0%). Patients who were taking steroids, anticancer agents, or antidepressants were excluded because those drugs are known to affect appetite, dietary content, and body composition.

### Assessment of dietary intake of fatty acids

We analyzed each patient’s dietary intake pattern of several fatty acids (palmitate, stearate, oleate, linoleic acid, arachidonate, and docosahexaenoic acid (DHA) during the month preceding analysis, using a brief self-administered diet history questionnaire (BDHQ), and compared the results among the three groups (patients of NYHA classes I, II, and III). The BDHQ is a 4-page structured questionnaire that calculates the consumption frequency of 58 selected food and beverage items based on the Standard Tables of Food Composition in Japan. The BDHQ has been validated to have very good correlation with a 150-item comprehensive diet history questionnaire [11]. The value of each item was energy-adjusted by density methods (g/1000 kcal) for each patient. The BDHQ was performed by two or more registered dietitians who were blinded to the patients’ clinical findings.”

### Establishment of myocardial infarction mice

Male C57BL/6 J mice (9–12-weeks old; CLEA Japan, Tokyo) were bred in a pathogen-free environment and housed in an animal room maintained at 23–25 °C under controlled conditions on a 12-h light/dark cycle. Diet and water were provided ad libitum.

The HF post-myocardial infarction model was established by ligating the left coronary artery as described [12, 13]. A sham surgery operation without ligation of the coronary artery was also performed as a control; the experiments were mainly performed 4 weeks after the surgery in the surviving control (*n* = 10) and HF (*n* = 10) mice. For each surgical procedure, the mice were anesthetized with an intraperitoneal injection (i.p.) of a mixture of 0.3 mg/kg of medetomidine (Dorbene®, Kyoritsuseiyaku, Tokyo), 4.0 mg/kg of midazolam (Dormicum®, Astellas Pharma, Tokyo), and 5.0 mg/kg of butorphanol (Vetorphale®, Meiji Seika Kaisha, Tokyo). The adequacy level of anesthesia was monitored based on the disappearance of the pedal withdrawal reflex.

After 4 weeks, echocardiography was performed. The mice were then sacrificed, and the hearts were excised. Biochemical measurements, analysis of mitochondrial respiration, and immunoblotting including sodium dodecyl sulfate-polyacrylamide gel electrophoresis (SDS-PAGE) or blue native (BN)-PAGE were performed.

### Linoleic acid administration to mice

Once mice developed HF after their myocardial infarction surgery, they were divided into two groups; one group was fed a normal-diet (CE-2; CLEA Japan, *n* = 34) and the other group was fed a linoleic acid-rich diet (*n* = 12) until their sacrifice 4 weeks after surgery. The linoleic-acid diet was prepared as described previously [14]. It contained the following ingredients in weight%: 20 casein, 5 high-linoleate safflower oil, 13.2 α-cornstarch, 10 sucrose, 5 cellulose, 3.5 mineral mixture, 1 vitamin mixture, 0.3 L-cysteine, 0.25 choline bitartrate, 0.0014 tert-butylhydroquinone, and 41.75 cornstarch. After 4 weeks, echocardiography was performed, the mice were sacrificed, and their hearts were excised. Biochemical measurements, evaluation of mitochondrial respiration, and immunoblotting, including SDS-PAGE and BN-PAGE were performed.

### Echocardiography

Standard echocardiographic short-axis views were obtained at the levels of the papillary muscles under light anesthesia using tribromoethanol/amylene hydrate (avertin; 2.5% wt/vol, 8 μL/g body weight, i.p.). The left ventricular (LV) end-diastolic diameter, LV end-systolic diameter, and LV fractional shortening from M-mode frames at a paper speed of 50 mm/sec were measured [12, 15].

### Preparation of isolated mitochondria from mouse heart

Mitochondria were isolated from mouse hearts. The heart was quickly harvested, and mitochondria were isolated as described previously [16–18]. With regard to the LVs from HF mice, infarct areas were excluded, and only non-infarct areas were used for the experiments. Briefly, heart tissues were minced on ice and then incubated for 2 min in mitochondrial isolation buffer (100 mmol/L sucrose, 100 mmol/L KCL, 1 mmol/L KH_2_PO_4_, 0.1 mmol/L EGTA, 0.2% bovine serum albumin, and 50 mmol/L Tris-HCl; pH 7.4) containing 0.1 mg/mL proteinase (Sigma-Aldrich, St. Louis, MO). Tissues were gently homogenized with six strokes using a motor-driven Teflon pestle in a glass chamber.

Homogenates were centrifuged at 700 *g* for 10 min. Supernatants were centrifuged at 10,000 *g* for 10 min, and pellets were washed and centrifuged at 7000 *g* for 3 min. The final pellets were suspended in suspension buffer containing 225 mmol/L mannitol, 75 mmol/L sucrose, 10 mmol/L Tris, and 0.1 mmol/L EDTA, pH 7.4. Finally, mitochondrial protein concentrations were measured by the bicinchoninic acid assay. These samples were used to measure mitochondrial function, for immunoblotting (SDS-PAGE and BN-PAGE), and lipidome analysis.

### Cell culture and knockdown of CRLS1

H9c2 (ATCC® CRL-1446™) cells were maintained in Dulbecco’s Modified Eagle’s Medium (4.5 g glucose/L; Sigma) containing 10% fetal bovine serum (Cell Culture Bioscience), 100 IU/mL penicillin, and 100 IU/mL streptomycin in a humidified incubator (Sanyo, Osaka, Japan) at 37 °C with 5% CO_2_ in air. H9c2 cells were plated in a T75 flask at a density of 1.0–1.6 × 10^6^ cells and then transfected 24 h later with 25 or 50 nmol of a nontargeting pool of small interfering RNA (siRNA) or siRNA against cardiolipin synthase 1 (*CRLS1*, Dharmacon, Brébières, France) using DharmaFECT reagent (Dharmacon). At 72–96 h after transfection, the cells were harvested and lysed with homogenization buffer containing 10 mM HEPES-KOH (pH 7.4), 220 mM mannitol, and 0.07 M sucrose with several deacetylase inhibitors. The mitochondrial fraction was also isolated by the sequential centrifugation method [19].

### Immunoblotting

Immunoblotting was performed as described previously [20]. Briefly, mitochondrial protein samples were separated by SDS-PAGE and transferred to a polyvinylidene fluoride (PVDF) membrane (Bio-Rad, Hercules, CA). The membrane was blocked for 1 h at room temperature in Tris-buffered saline containing 0.1% Tween 20 (TBS-T) buffer containing 3% milk, and then incubated with the primary antibodies (CRLS1 and total OXPHOS antibody cocktail; Abcam, Cambridge, MA) at a dilution of 1:1000 overnight at 4 °C. After three washes with TBS-T, the membrane was incubated with a horseradish peroxide-conjugated secondary antibody at a dilution of 1:5000 for 1 h at room temperature. After washing, the membrane was developed with ECL, ECL Prime, or SuperSignal West Dura Reagent (GE Healthcare Life Sciences, Piscataway, NJ) and then processed for detection with ChemiDoc XRS+ (Bio-Rad). The density of the band signals was quantified with Image J software (U.S. National Institutes of Health, Bethesda, MD). The expression levels of proteins are shown as values corrected with total protein (Coomassie brilliant blue [CBB] staining) of mitochondrial samples.

### BN-page

BN-PAGE was performed as described previously [21]. Briefly, mitochondrial proteins were extracted with 5% digitonin (Invitrogen, Carlsbad, CA; protein:detergent ratio of 1:10) and 4× buffer (Invitrogen) on ice for 30 min. After centrifugation at 10,000 *g* for 10 min at 4 °C, the supernatants were collected. The remaining lysates were combined with Coomassie blue G-250 dye (Invitrogen; protein:detergent ratio of 1:10) and added to 3–12% NativePAGE Novex Bis-Tris Gel (Invitrogen), then separated by electrophoresis using Anode and Cathode buffer (Invitrogen) at 10 mA for 1 h and at 150 V for 2 h on ice. Protein complexes in the samples after electrophoresis were denatured using denaturing buffer (20 mmol Tris, 200 mmol glycine, and 1% SDS). The gels were then transferred by electroblotting to PVDF membranes (Bio-Rad) using transfer buffer at 25 V for 2 h.

### Measurement of mitochondrial OXPHOS capacity

Mitochondrial respiratory capacities were measured in isolated mitochondria at 37 °C with a high-resolution respirometer (Oxygraph-2 k; Oroboros Instruments, Innsbruck, Austria) [21–23]. The respirometer chamber was filled with 2 mL of MiR05 medium, and then isolated mitochondria (approx. 30–100 μg) were added, followed by the substrate ADP, and inhibitors in the following order: malate (2 mmol/L), pyruvate (5 mmol/L), glutamate 10 mmol/L, MgCl_2_ (3 mmol/L), ADP (10 mmol/L; to measure CI respiration), succinate (10 mmol/L; to measure CI + II respiration), and rotenone (0.5 μmol/L; to measure complex II respiration). O_2_ consumption rates (i.e., respiratory rates) are expressed as the O_2_ flux normalized to the mitochondrial protein concentration (μg/μL). DatLab software (Oroboros Instruments) was used for data acquisition and data analysis.

### Lipidome (phospholipid fatty acids, including cardiolipin) analysis by liquid chromatography-mass spectrometry (LC-MS)

To perform lipidome analysis, we analyzed 5 μL of the supernatant by LC-MS using Nexera X2 (Shimadzu Corp., Kyoto, Japan) coupled with a Q-Exactive mass spectrometer (Thermo Fisher Scientific, San Jose, CA), followed by separation by reverse-phase LC using an Accucore C18 column (150 × 2.1 mm, 2.6 μm, Thermo Fisher Scientific). The mobile phase consisted of H_2_O:acetonitrile (60:40, v/v) in 10 mM ammonium formate and 0.1% formic acid (A) and isopropanol:acetonitrile (90:10, v/v) in 10 mM ammonium formate and 0.1% formic acid (B). The column oven temperature was 40 °C. The gradient elution program was as follows: a flow rate of 0.3 mL/min: 0–1.5 min, 32% B; 1.5–6 min, 32–58% B; 6–18 min, 58–75% B; 18–21 min, 75–97% B; 21–25 min, 97% B; 25.1–35 min, 32% B. The parameters for the heated electrospray ionization source in negative-ion mode were as follows: flow rates of the sheath gas and auxiliary gas were 40 and 10 (arbitrary units), respectively. The spray voltage, capillary temperature, and heater temperature were maintained at 2.5 kV, 350 °C, and 300 °C, respectively. The S-lens RF level was set at 50. The Q-Exactive mass spectrometer was operated at a resolving power of 70,000 in full-scan mode (scan range: 200–2000 m/z, automatic gain control target: 1e6); maximum ion injection time: 250 msec. Data processing was performed using LipidSearch software (Mitsui Knowledge Industry, Osaka, Japan), and ions corresponding to cardiolipin were subjected to statistical analysis.

### Statistical analysis

Data of the dietary intake of fatty acids, demographic factors, causes of heart failure, medications, and echocardiographic parameters in HF patients were summarized as the mean ± standard deviation (SD). One-way ANOVA was used to compare continuous variables. Categorical variables are presented as numbers or percentages, and were compared by the chi-squared test. All analyses were performed using JMP Pro 14.0 (SAS Institute, Cary, NC,). Other data were expressed as the mean ± standard error of the mean (SE). Statistical analyses were performed using the Student *t*-test for comparisons between two groups and one-way analysis of variance followed by the Tukey test for three groups with GraphPad Prism 6 software (GraphPad, San Diego, CA). A univariate linear regression model was used to determine the correlation between two variables. A *P*-value less than 0.05 was considered to indicate a statistically significant difference between two groups.

## Results

### Linoleic acid intake reduces in HF patients

**Fig. 1:**
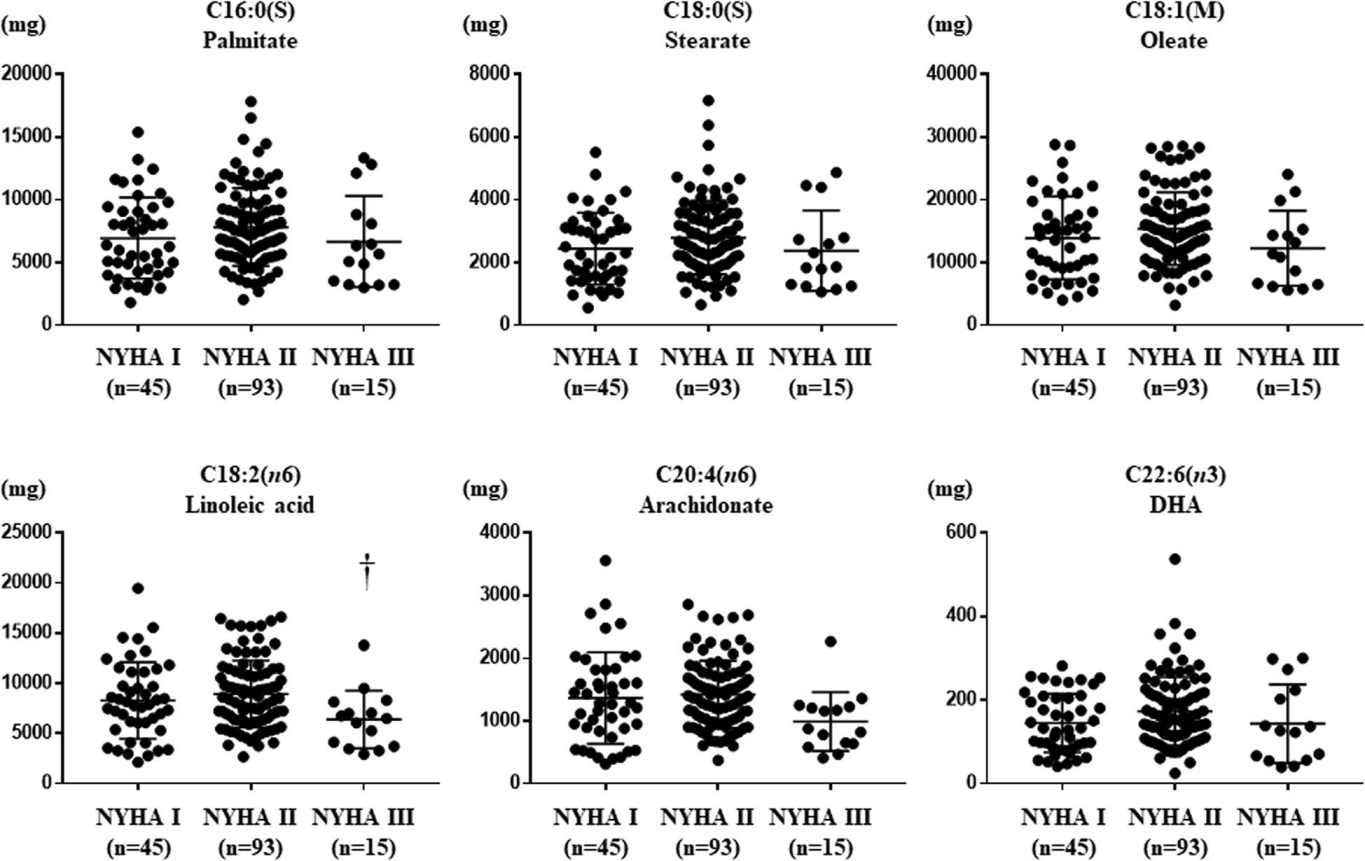
Linoleic acid intake was significantly lower in NYHA III patients compared with NYHA II patients. The dietary intake of palmitate, stearate, oleate, linoleic acid, arachidonate, and docosahexaenoic acid (DHA) in heart failure (HF) patients of NYHA class I (*n* = 45), II (*n* = 93), and III (*n* = 15). Data are shown as the mean ± SD. †*p* < 0.05 vs NYHA II. NYHA, New York Heart Association

We first compared the dietary intake of each fatty acid among HF patients of NYHA class I (*n* = 45), II (*n* = 96), and III (*n* = 15). Table 1 shows demographic factors, causes of heart failure, medications, and echocardiographic parameters in each NYHA class. Rate of men among all patients and the use of an ACE inhibitor or ARB as medication were significantly different among the three groups (Table 1). On the other hand, there were no differences in other data, including the cause of heart failure and cardiac function (Table 1). Among the several fatty acids that we analyzed, the dietary intake of linoleic acid was significantly lower in NYHA III patients than in NYHA II patients (Fig. 1). A previous study demonstrated that cardiolipin content was decreased in the cardiac mitochondria of aged spontaneous hypertensive HF rats, and linoleic acid is the major fatty acid moiety of cardiolipin, as mentioned earlier [6]. We were thus interested in further studying the possible association between the dietary intake of linoleic acid and the cardiolipin content of cardiac mitochondria, particularly with regard to cardiac mitochondrial function.

**Table 1:** Baseline patient characteristics

	NYHA I	NYHA II	NYHA III	*P*-value
Number	45	93	15	
Demographic factors				
Age, yrs	66 ± 12	68 ± 13	67 ± 18	0.734
Men, n (%)	34 (76)	73 (71)	5 (33)	0.010
Body mass index, kg/m^2^	23.7 ± 3.3	23.7 ± 5.6	23.3 ± 5.8	0.685
Cause of heart failure, n (%)				
Ischemic heart disease	16 (36)	23 (25)	2 (13)	0.264
Dilated cardiomyopathy	14 (31)	5 (5)	7 (47)	0.393
Valvular heart disease	11 (24)	6 (6)	6 (40)	0.396
Hypertensive heart disease	12 (27)	27 (29)	4 (27)	0.965
Hypertrophic cardiomyopathy	0 (0)	3 (3)	0 (0)	0.189
Medication, n (%)				
β-blocker	34 (76)	84 (90)	12 (80)	0.403
ACE-I or ARB	28 (62)	76 (82)	11 (73)	0.029
Diuretics	34 (76)	72 (77)	14 (93)	0.311
Echocardiographic parameters				
LVEDD, mm	55 ± 9	57 ± 12	62 ± 13	0.089
LVEF, %	46 ± 14	45 ± 16	35 ± 16	0.057

NYHA class, New York Heart Association class; ACE-I, angiotensin converting enzyme inhibitor; ARB, angiotensin II receptor blocker; LVEDD, left ventricular end-diastolic diameter; LVEF, left ventricular ejection fraction. Data are expressed as means ± SD

### Heart mitochondrial cardiolipin and function reduce in HF mice

**Fig. 2:**
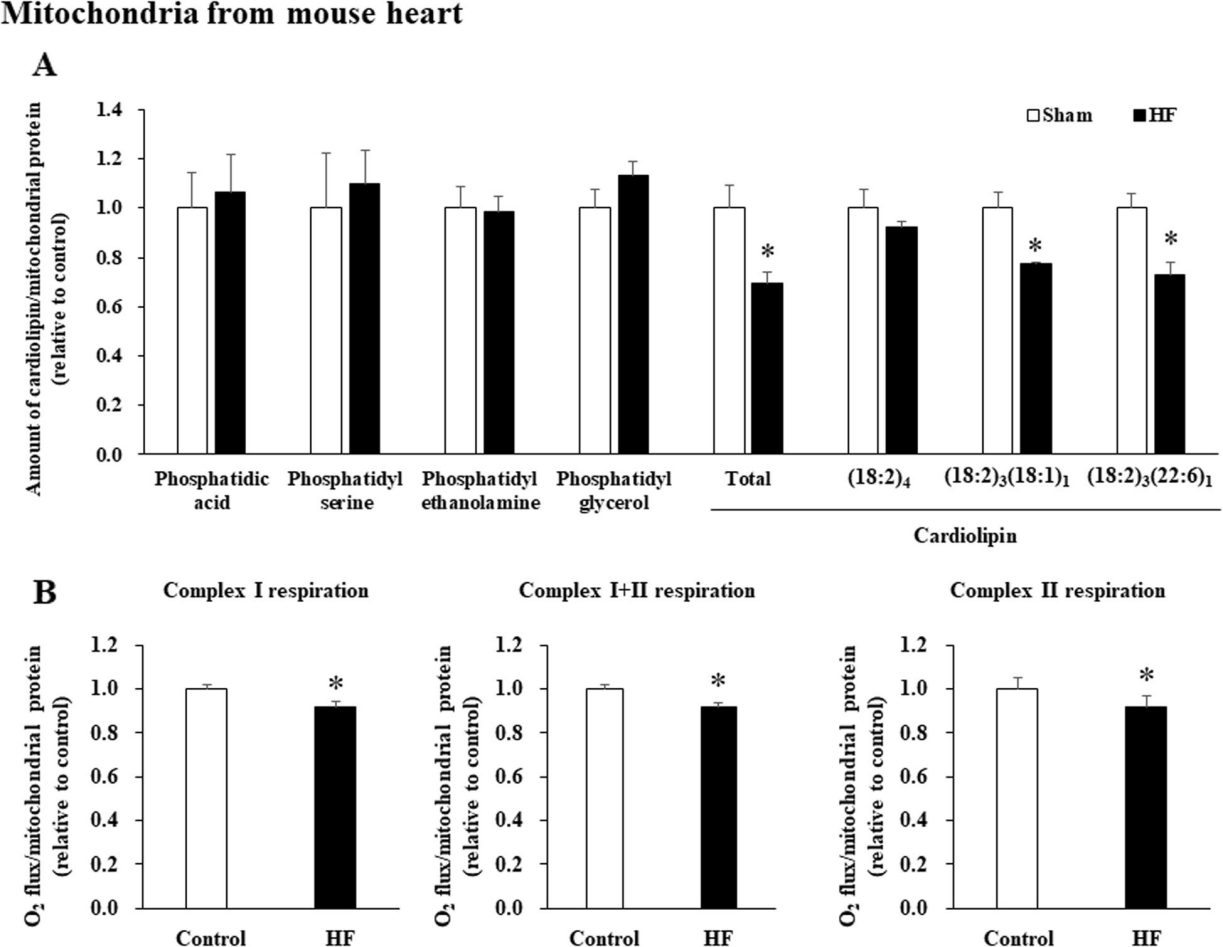
Cardiolipin content and mitochondrial respiration were decreased in HF mice. **a**. Comparison of phospholipid fatty acids (phosphatidic acid, phosphatidyl serine, phosphatidyl ethanolamine, and phosphatidyl glycerol) and cardiolipin (total, (18:2)_4_, (18:2)_3_(18:1)_1_, (18:2)_3_(22:6)_1_) content in isolated heart mitochondria between control (*n* = 4) and HF mice (*n* = 4). **b**. Complex I-linked respiration, complex I + II-linked respiration, and complex II-linked respiration in heart mitochondria per mitochondrial protein in control (*n* = 4) and HF mice (*n* = 4). Data are shown as the mean ± SE. *p < 0.05 vs control mice

Consistent with a previous report [6], we then found that levels of phospholipid fatty acids (phosphatidic acid, phosphatidyl serine, phosphatidyl ethanolamine, and phosphatidyl glycerol) do not differ between a mouse model of HF after myocardial infarction and control mice, whereas the total cardiolipin content, including the molecular species composition of cardiolipin [(18:2)_3_(18:1)_1_ and (18:2)_3_(22:6)_1_] in cardiac mitochondria, is significantly lower in HF mice [12–14] than in control mice (Fig. 2a). In cardiac mitochondria of these HF mice, both CI-linked respiration (CI + III + IV) and complex II-linked respiration (CII + III + IV) per mitochondrial protein, as well as CI + II-linked respiration were significantly reduced compared with control mice (Fig. 2b).

Similar to our earlier studies, we herein confirmed using these HF mice that cardiac function (%fraction shortening), as analyzed by echocardiography, was significantly decreased and that the LV was larger than control mice at 4 weeks after surgery (control [*n* = 4] vs HF [*n* = 5]: %FS (%), 45.5 ± 0.9 vs 12.9 ± 3.4, *p* < 0.05; LV end-diastolic diameter, (mm), 3.25 ± 0.14 vs 5.04 ± 0.26, *p* < 0.05; LV end-systolic diameter (mm), 1.78 ± 0.10 vs 4.42 ± 0.39, *p* < 0.05).

### Assembly of supercomplexes and complex II correlates with cardiolipin content in mitochondria isolated from siCRLS1-treated cardiomyocytes

**Fig. 3:**
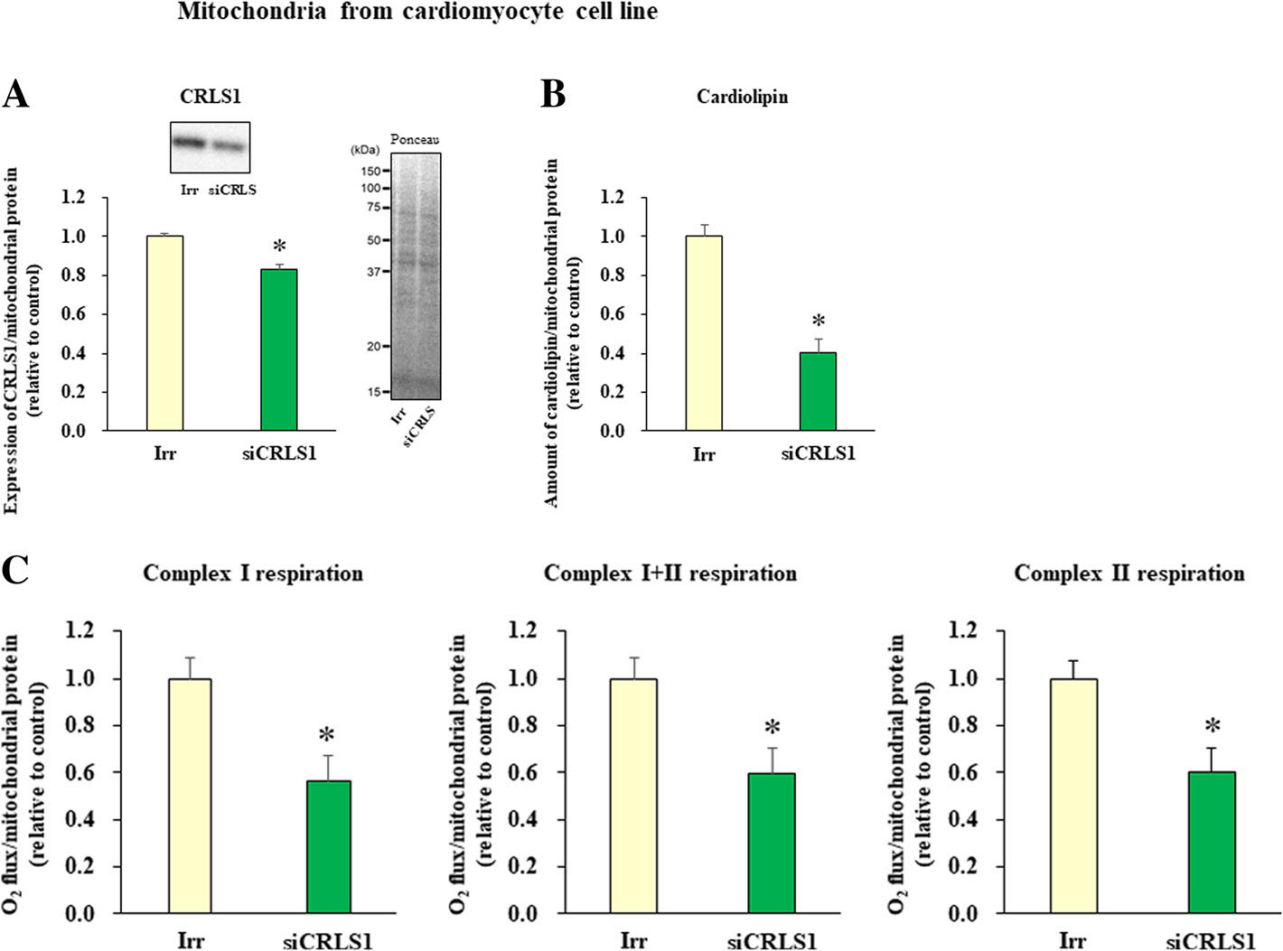
Cardiolipin content and mitochondrial respiration were decreased in cardiomyocytes by siRNA-mediated blockade of cardiolipin synthase 1 (*CRLS1*). Assays were started at 72 h after the transfection. **a**. Amount of CRLS1 in isolated mitochondria from cardiomyocytes treated with irrelevant siRNA (Irr) (*n* = 6) or *siCRLS1* (n = 6) (*left*). Total protein levels shown by Ponceau staining were used as the internal controls (*right*). The sizes of the molecular weight markers are indicated on the left in kDa. **b**. Cardiolipin content in isolated mitochondria from cardiomyocytes treated with an irrelevant siRNA (Irr, *n* = 5) or *siCRLS1* (*n* = 5). **C**. Complex I-linked respiration, complex I + II-linked respiration, and complex II-linked respiration in isolated mitochondria from cardiomyocytes treated with control (*n* = 4) and *siCRLS1* (*n* = 4). Data are shown as the mean ± SE. *p < 0.05 vs Irr

**Fig. 4:**
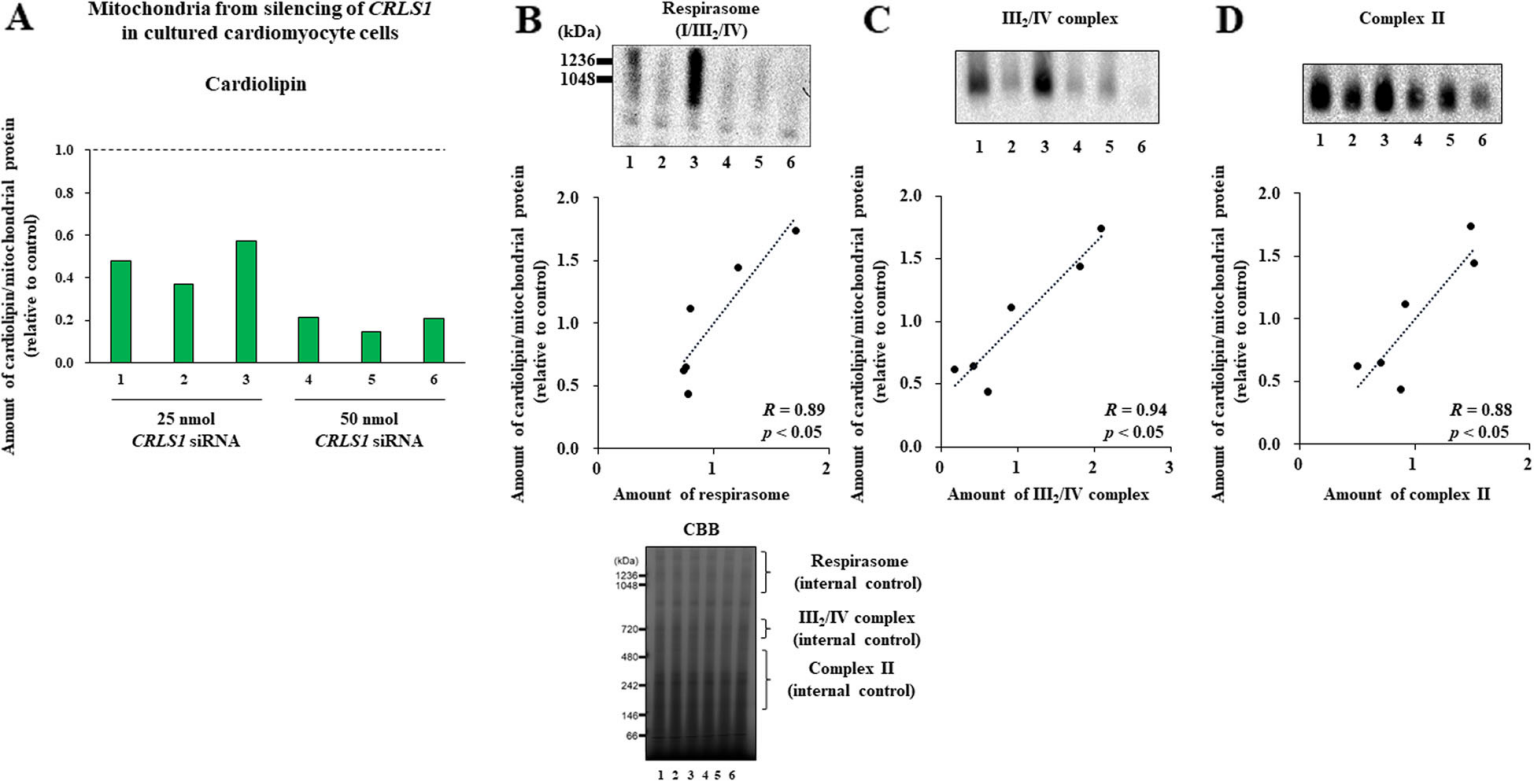
Amount of supercomplexes and complex II significantly correlated with cardiolipin content in mitochondria isolated from *siCRLS1*-treated cardiomyocytes. Assays were started at 96 h after the transfection with 25 or 50 nmol siRNA (*n* = 3 each). **a**. The cardiolipin content in mitochondria isolated from *siCRLS1* cardiomyocytes are shown relative to Irr (n = 6). **b**. Correlation between cardiolipin content and the amount of respirasomes (I/III_2_/IV_n_). **c**. Correlation between cardiolipin content and the amount of complex III_2_/IV. **d**. Correlation between cardiolipin content and the amount of complex II. Pearson correlation coefficients (R) and *p*-values are shown

The above results suggested a possible link between the reduced amounts of cardiolipin and the reduced assembly of the mitochondrial OXPHOS components in cardiomyocytes. We then confirmed this association using cultured rat H9c2 cardiomyocytes [24]. Silencing of *CRLS1*, which encodes cardiolipin synthesis 1, by the siRNA method significantly reduced cardiolipin levels in these cells (Fig. 3a,b). In *siCRLS1* cells, CI-linked, CII-linked, as well as CI + II-linked mitochondrial respiration were significantly reduced compared with irrelevant siRNA-treated control cells (Fig. 3c). We next varied the concentration of siRNA (25 or 50 nmol), which resulted in differences in *CRLS1* expression levels and cardiolipin concentrations (see Fig. 4a). Taking advantage of such variations, we then analyzed the association between cardiolipin content and the assembly of OXPHOS components. We found significant correlations between cardiolipin content per mitochondrial protein and the amounts of the CI/III_2_/IV respirasome and CIII_2_/IV-supercomplex, as well as CII-subunit assembly in mitochondria isolated from these cells (Fig. 4b-d). Therefore, these results collectively indicated that a reduction in cardiolipin levels is closely associated with reduced mitochondrial respiration in cardiomyocytes, as has been implicated in various types of cells and organisms as mentioned above.

### Supplementation of linoleic acid improves heart mitochondrial crisis and cardiac dysfunction in HF mice

**Fig. 5:**
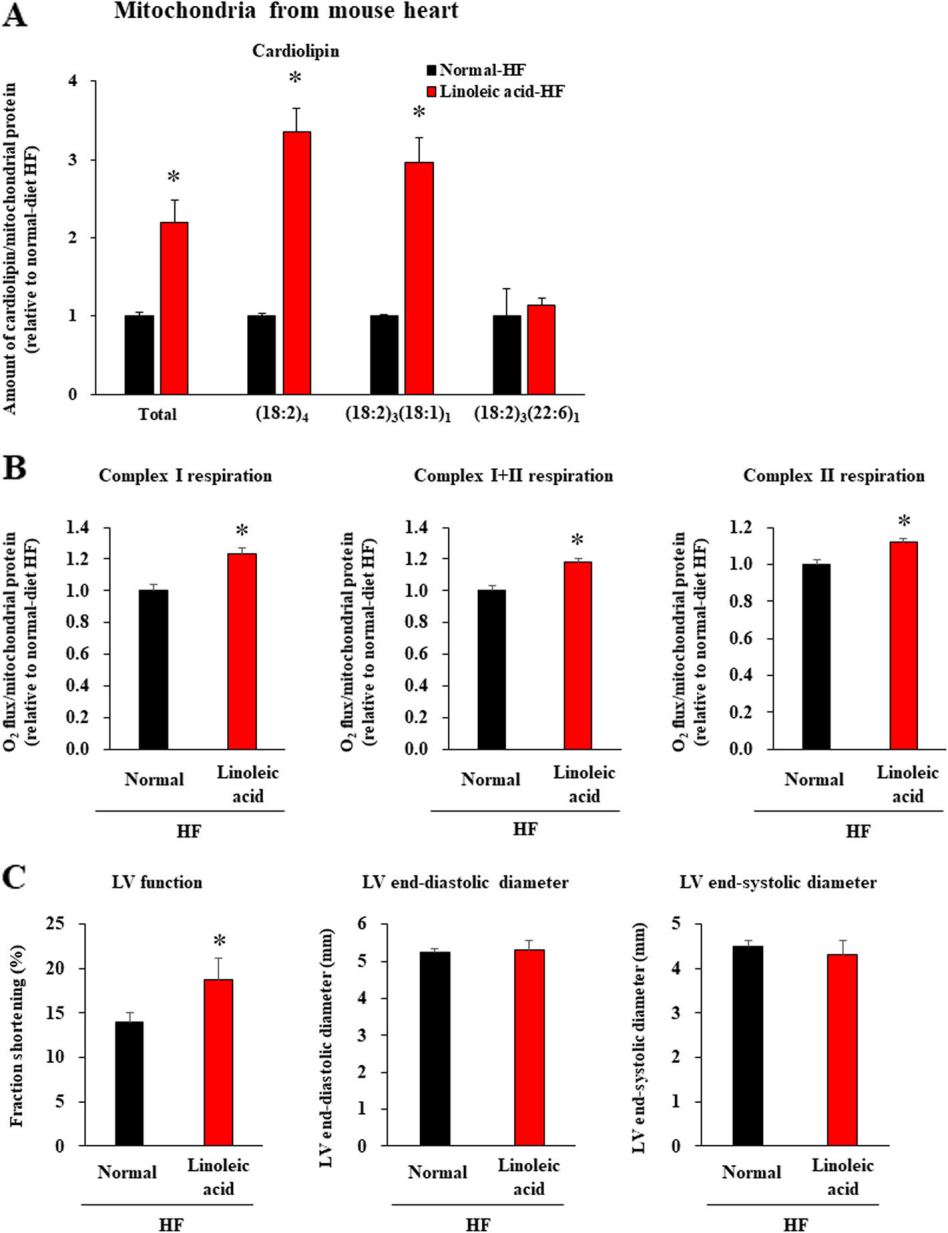
Supplementation of linoleic acid to HF mice increased cardiolipin content and improved mitochondrial respiration and cardiac function. **a**. Cardiolipin contents (total, (18:2)_4_, (18:2)_3_(18:1)_1_, (18:2)_3_(22:6)_1_) in isolated heart mitochondria from HF mice fed a normal diet (*n* = 5) and HF mice fed a linoleic acid-rich diet (*n* = 5). **b**. Complex I-linked respiration, complex I + II-linked respiration, and complex II-linked respiration per heart mitochondrial protein in HF mice (*n* = 5) and HF mice fed a linoleic acid-rich diet (*n* = 5). **c**. LV function, LV end-diastolic diameter, and LV end-systolic diameter in HF mice fed a normal diet (*n* = 25) and HF mice fed a linoleic acid-rich diet (*n* = 8). Data are shown as the mean ± SE. LV, left ventricle. *p < 0.05 vs HF mice on a normal diet”

**Fig. 6:**
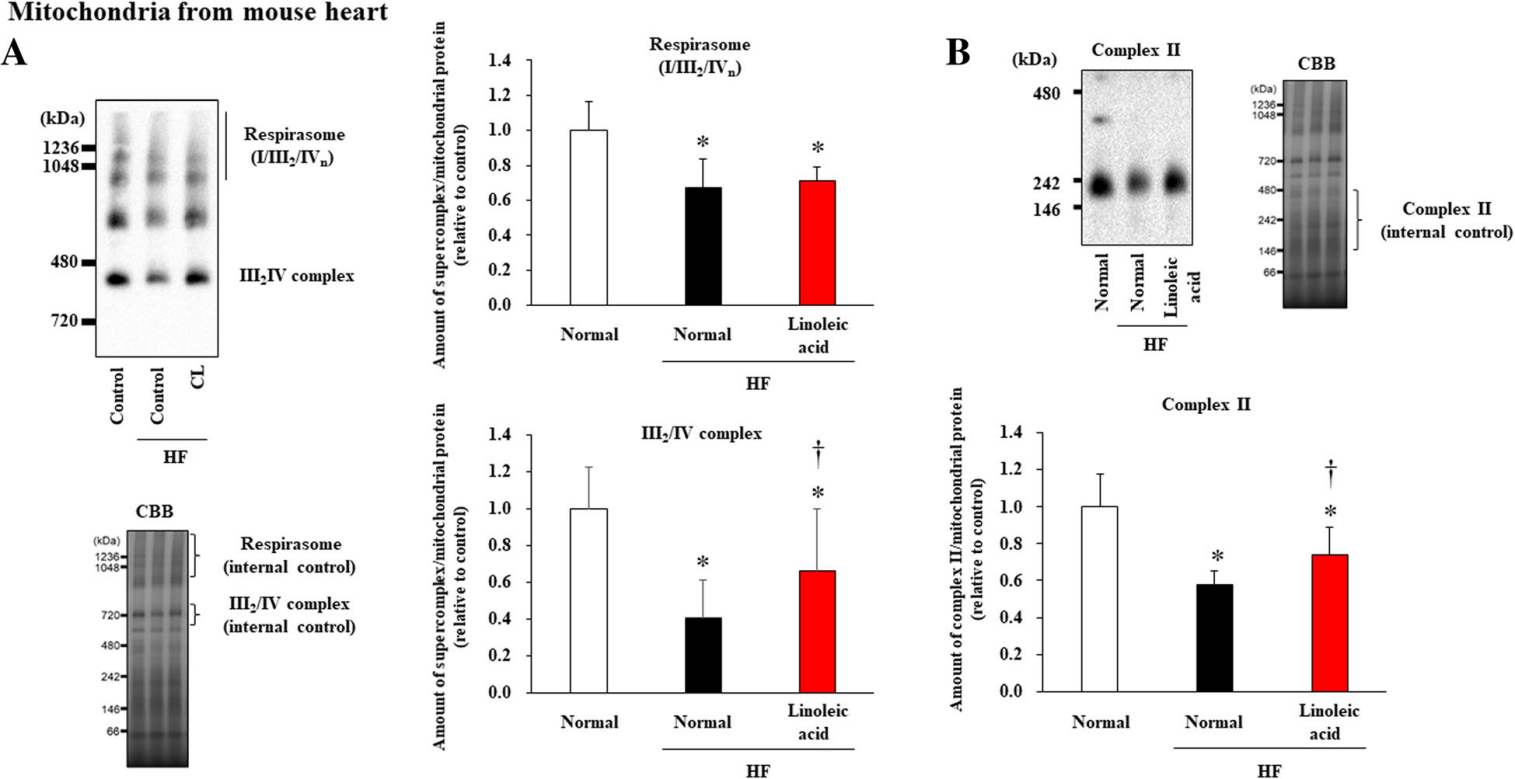
Respirasome amounts were decreased in HF mice and not restored by linoleic acid, whereas amounts of complex III_2_/IV and complex II were partially restored by linoleic acid supplementation. **a**. *Top*: Amounts of supercomplexes (respirasome and complex III_2_/IV) analyzed by BN-PAGE in mice fed a normal diet (*n* = 4), and HF mice fed a normal diet (*n* = 4) and linoleic acid-rich diet (*n* = 4). Total protein levels shown by CBB staining (*bottom*) were used as internal controls. The sizes of the molecular weight markers are indicated on the left in kDa. **b**. Amounts of complex II analyzed by BN-PAGE in mice fed a normal diet (n = 4), and HF mice fed a normal diet (n = 4) and a linoleic acid-rich diet (n = 4). Total protein levels shown by CBB staining were used as internal controls. The sizes of the molecular weight markers are indicated on the left in kDa. Data are shown as the mean ± SE. *p < 0.05 vs mice fed a normal diet. †*p* < 0.05 vs. HF mice fed a normal diet

We then sought to investigate the associations among the dietary intake of linoleic acid, cardiolipin levels in cardiac mitochondria, and CI/CII-linked respiration of cardiomyocytes in vivo. We fed HF model mice with a diet supplemented with a high amount of linoleic acid (about 150 μg/day), and measured cardiolipin content and OXPHOS activities in their heart mitochondria after 4 weeks on a continued linoleic acid diet. Total cardiolipin content, including the molecular species composition of cardiolipin [(18:2)_4_ and (18:2)_3_(18:1)_1_], in cardiac mitochondria were significantly improved by linoleic acid supplementation, to levels almost equal to those of healthy mice (Fig. 5a and Fig. 2a). This supplementation also improved both complex I-linked and complex II-linked respiration of cardiac mitochondria (Fig. 5b) and LV function (Fig. 5c).” On the other hand, contrary to cultured cardiomyocytes, we did not observe any notable improvement in CI/III_2_/IV respirasome supercomplex formation in these HF mice fed linoleic acid, whereas assembly of the CIII2/IV supercomplex and CII subunits were indeed significantly improved (Fig. 6a,b).

## Conclusions

Linoleic acid is an essential omega-6 fatty acid, and a prospective study demonstrated that a high conjugated linoleic acid concentration is associated with a lower risk of HF incidence in older men [25], whereas its excess intake is well known to be harmful to human health, including the increased risk of arteriosclerosis [26]. Ironically, however, we here found that HF patients who take less amounts of linoleic acid in their daily diets have more severe HF. Furthermore, we demonstrated that both CI-linked and CII-linked respiration of heart mitochondria are affected by HF, and that dietary intake of linoleic acid improves both of these respiratory mechanisms, primarily through an improvement in the assembly of CII subunits and the CIII_2_/CIV supercomplex.

In our animal experiments, dietary intake of linoleic acid did not improve the assembly of the CI/III_2_/IV respirasome, whereas in cultured cells, cardiolipin content was statistically associated with the assembly of the CI/III_2_/IV respirasome, CII-subunits, and the CIII_2_/CIV supercomplex. Therefore, it is likely that dysregulation of respirasome assembly that occurs in HF cannot be easily repaired by the enhanced intake of linoleic acid or by enhanced cardiolipin content. We have no clear explanation for this, but it is possible that this phenomenon is simply a reflection of the possible ordered assembly of OXPHOS components. Nevertheless, whether other factors or events that are involved in respirasome assembly, or other aspects of mitochondrial function, are specifically affected by HF should be further investigated. The mechanisms by which cardiolipin content are reduced by HF, and whether cardiolipin content can be restored to a certain extent in HF mice by linoleic acid supplementation, also needs to be clarified.

We hence concluded that dietary supplementation of linoleic acid is beneficial for improving cardiac malfunction in HF, but is unable to completely cure HF.

## Data Availability

The datasets generated during and/or analyzed during the current study are available from the corresponding author upon reasonable request.

## Abbreviations

BDHQ: brief self-administered diet history questionnaire
BN-PAGE: Blue Native-Polyacrylamide Gel Electrophoresis
CBB: Coomassie Brilliant Blue
CI: complex I
CII: complex II
CRLS1: cardiolipin synthase 1
HF: heart failure
LA: linoleic acid
LV: left ventricle
NYHA: New York Heart Association
OXPHOS: oxidative phosphorylation
